# Analysis of fungal community structure and co-occurrence networks across vegetation types in volcanic lava habitats

**DOI:** 10.3389/ffunb.2026.1760883

**Published:** 2026-02-24

**Authors:** Jiahui Cheng, Lihong Xie, Mingyue Jiang, Jiaxin Xue, Hongjie Cao, Yulin Guo, Fan Yang, Qingyang Huang

**Affiliations:** 1 Institute of Natural Resources and Ecology, Heilongjiang Academy of Sciences, Harbin, Heilongjiang, China; 2 School of Life Science and Technology, Mudanjiang Normal University, Mudanjiang, Heilongjiang, China

**Keywords:** co-occurrence network analysis, functional prediction, fungal community structure, lava habitats, vegetation types

## Abstract

The eruption of Wudalianchi Volcano directly damaged the soil and vegetation, forcing succession to restart from bare land. It influenced subsequent vegetation succession. This study utilizes the Wudalianchi volcanic lava plateaus as a model, employing high-throughput sequencing to unravel the drivers of soil fungal diversity across a vegetation gradient: moss (M), herb (H), shrub (S), broadleaf forest (B), and mixed coniferous-broad-leaved forest (C). This study found that *Ascomycota* (43.39%–71.54%) and *Basidiomycota* (5.36%–53.21%) were the dominant phyla. *Ascomycota* peaked in the C community, whereas *Basidiomycota* was most abundant in the M community. At the genus level, *Cortinarius*, *Mortierella*, and *Scleroderma* dominated in the B, H, and M communities, respectively. For fungal communities, Shannon and Chao indices followed the order: S > H > C > M > B. Co-occurrence network analysis indicated the greatest complexity and connectivity in the S community, which had the most nodes, links, and the highest average degree. Fungal functional guilds shifted across the gradient: symbiotrophic fungi prevailed in the B and M, while saprotrophic fungi dominated H and C communities. Soil physicochemical properties were the primary determinants of fungal community structure and function. In conclusion, significant differences exist in the structure, diversity, and function of soil fungal communities across different vegetation types in volcanic lava habitats. Soil TP, pH, and N/P ratio were identified as key drivers, with shrub vegetation playing a critical role in fostering complex fungal networks and functional balance. This study underscores the key regulatory role of specific soil properties and vegetation succession in shaping fungal communities, providing a framework for understanding microbial assembly in extreme environments.

## 1 Introduction

Volcanic lava habitats are among earth’s most distinctive extreme habitats. Eruptions deposit nutrient-poor ash–formed from rapidly cooled and fragmented magma–creating edaphic stresses such as low organic matter, extreme pH, and limited water-holding capacity (Setiawati et al., 2024). Volcanic ash can release nutrients upon weathering, and accumulating plant litter drives soil development, forming heterogeneous microhabitats for microbial colonization (Duarte et al., 2024). Extreme environments provide unique niches that test microbial limits while selecting for novel adaptations, offering ideal models for studying microbial ecology and evolution (Chen et al., 2024). Changes in the fungal community composition and diversity during forest vegetation succession are key indicators of variation in ecosystem functions (Deng et al., 2019). Volcanic lava habitats typically undergo well-predictable vegetation succession, from pioneer mosses and herbs to shrubs and forest, with each stage uniquely shaping soil properties (Kulkarni et al., 2022). In contrast, primary succession on volcanic bare sand bypasses the herbaceous and shrub stages, leading to the establishment of forests dominated by pioneer trees such as *Betula platyphylla* and *Larix gmelinii* (Zhou et al., 2016). This sequential progression provides a natural gradient to examine how fungal communities adapt to changing soils and how these adaptations, in turn, support plant establishment and ecosystem restoration.

Soil microorganisms, primarily bacteria and fungi, are fundamental to terrestrial ecosystem function. They drive nutrient cycling, promote soil structure and fertility, and help maintain plant nutritional homeostasis through diverse functional roles (Cui et al., 2024). Fungi, in particular, act as key ecosystem agents by decomposing organic matter, facilitating plant nutrient acquisition, and forming extensive mycorrhizal networks that enhance host plant fitness (Crowther et al., 2016). The structure and dynamics of these fungal communities are largely shaped by plant-soil feedbacks, where vegetation alters soil properties–such as pH, carbon-to-nitrogen ratio, and nutrient availability–which in turn select for distinct fungal taxa (Sui et al., 2022). Consequently, the composition and functional diversity of soil fungi are critical for maintaining soil health and driving vegetation succession (Cui et al., 2024). However, despite their ubiquity, the distribution patterns of fungal phyla and functional groups, especially their responses to vegetation-driven changes in extreme environments, remain poorly understood (Hagedorn et al., 2019). Shifts in fungal community composition and diversity during vegetation succession are robust indicators of broader ecosystem transitions, underscoring their central role in ecological recovery (Chen et al., 2019; Chen W.Z et al., 2023). Despite the critical importance of fungi in biogeochemical cycling and ecosystem recovery, their community dynamics in volcanic systems remain poorly resolved.

To address these knowledge gaps, we conducted a study in the Wudalianchi volcanic lava soil in China’s Heilongjiang Province–an ideal system featuring well-defined vegetation succession gradients and distinct volcanic soils. We investigated the interactions among vegetation types, soil properties, and fungal communities to answer three key questions: (1) How do fungal community composition, diversity, and functional guilds respond to these vegetation-driven soil changes? (2) Which soil factors are most important in structuring fungal communities? (3) How do these relationships underpin ecosystem restoration? Our findings provide new insights into vegetation-fungus-soil interactions in one of earth’s most extreme environments, with implications for leveraging microbial communities to enhance ecological recovery.

## 2 Materials and methods

### 2.1 Site description and soil sampling collection

**Figure 1:**
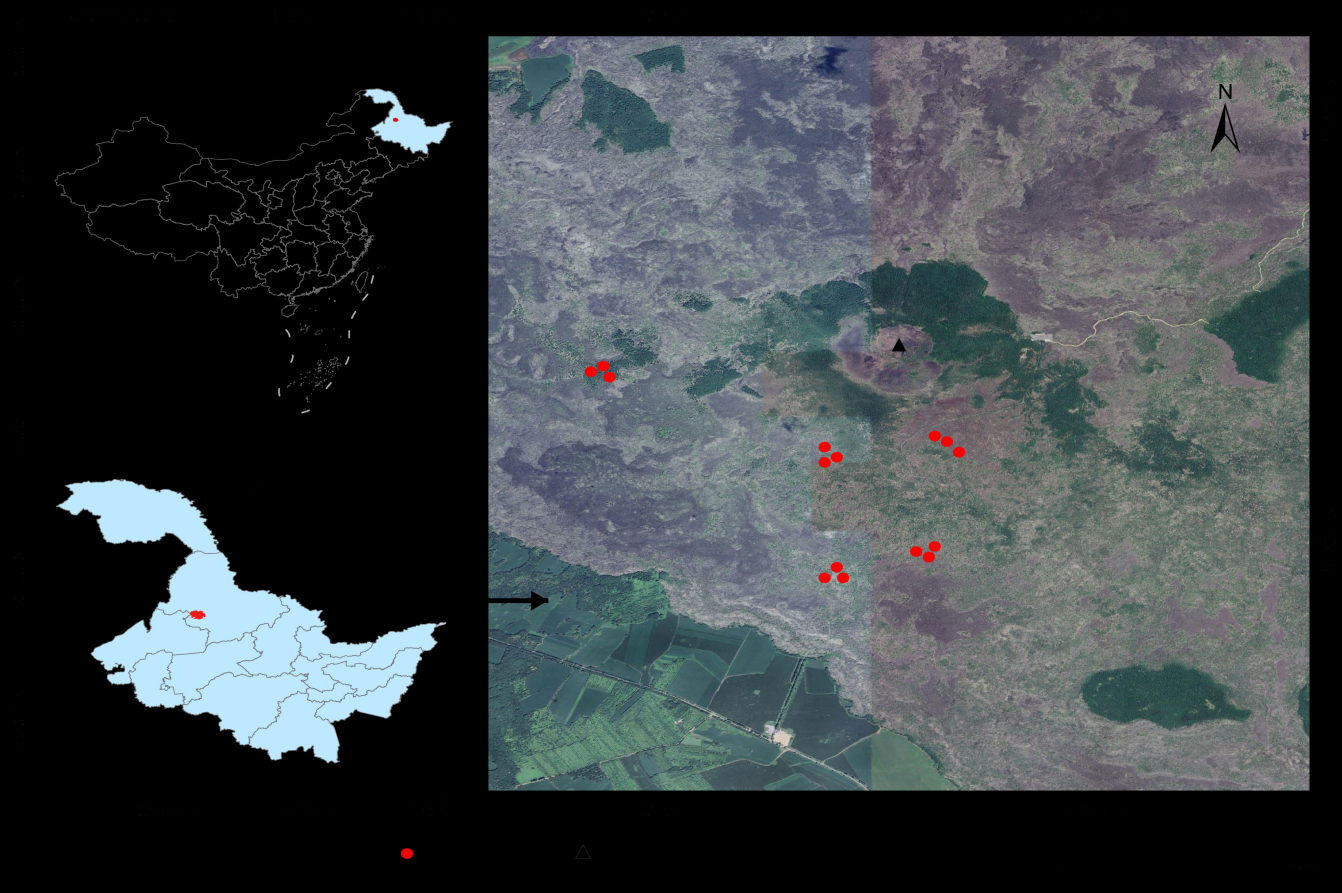
Geographical location of sampling sites in this study.

Our study area extends for 988 km^2^ in the Wudalianchi Volcano (125°45′-126°30′ E; 48°30′-48°50′ N), which is a well-preserved volcano in Heilongjiang Province, China (**Figure 1**). The climate is characterized by a continental monsoon pattern, with a mean annual temperature of -5 °C and annual precipitation averaging 476.33 mm.

Soil samples were collected in the selected area in August 2023. To ensure sampling uniformity, samples were collected from locations with similar elevation and soil types: moss, herb, shrub, broadleaf forest, and mixed coniferous-broadleaf forest. The basic information of sample plots across different vegetation types is presented in **Table 1**. Within the plots of each vegetation type, three 10 m × 10 m quadrats were established, with approximately 20 m spacing between adjacent quadrats. When sampling each plot, tree trunks and plot edges were avoided as much as possible. We removed surface litter and discarded stones and roots from the soil sample. Soil samples were collected from the 0–5 cm layer using the “five-point sampling method”. Soils from the same quadrat were thoroughly mixed to form a composite sample weighing approximately 1000 g. All final composite samples were transported to the laboratory for analysis. The collected soil samples were sieved through a 2 mm mesh to remove roots and leaves. One portion was stored at -80°C for DNA extraction, and the other portion was analyzed for soil physicochemical properties.

**Table 1:** Dominant species in different vegetation types.

Vegetation type	Dominant species	Coverage(%)	Altitude(m)
Moss	*Racomitrium canescens*	70-90	332-334
Herb	*Potentilla chinensis, Artemisia sacrorum, Patrinia rupestris* and *Rubus sachalinensis*	70-80	327-348
Shrub	*Sorbaria sorbifolia*	20-40	328-354
Broadleaf forest	*Populus davidiana, Betula platyphylla* and *P. koreana*	85-90	329-350
Mixed coniferous-broad-leaved forest	*Betula platyphylla, Larix gmelinii, Artemisia sacrorum*, and *Patrinia rupestris*	65-80	320-345

### 2.2 Determination of soil physical and chemical factors

The soil water content (WC) was determined using the drying method, soil pH (pH) was measured using a soil-water ratio of 1:5, and total nitrogen (TN) and total carbon (TC) were measured by the fully automated carbon and nitrogen analyzer Elementarvario ELIII (Elementar Analysensysteme GmbH, Langenselbold, Germany). Total phosphorus (TP) was determined using melted molybdenum, antimony, and scandium colorimetry.

### 2.3 DNA sequencing and bioinformatic analysis

Total microbial genomic DNA was extracted from soil samples using the E.Z.N.A.^®^ soil DNA kit (Omega Bio-tek, Norcross, GA, U.S.) according to manufacturer’s instructions. The quality and concentration of DNA were determined by 1.0% agarose gel electrophoresis and a NanoDrop 2000 spectrophotometer (Thermo Scientific, United States). The ITS region was amplified using primers ITS1F (5’-CTTGGTCATTTAGAGGAAGTAA-3’) and ITS2 (5’-GCTGCGTTCTTCATCGATGC-3’) by T100 Thermal Cycler PCR thermocycler (BIO-RAD, USA). The PCR reaction mixture including 4 μL 5 × Fast Pfu buffer, 2 μL 2.5 mM dNTPs, 0.8 μL each primer (5 μM), 0.4 μL Fast Pfu polymerase, 10 ng of template DNA, and ddH_2_O to a final volume of 20 µL. PCR amplification cycling conditions were as follows: initial denaturation at 95°C for 3 min, followed by 27 cycles of denaturing at 95°C for 30 s, annealing at 55°C for 30 s and extension at 72°C for 45 s, and single extension at 72°C for 10 min, and end at 4°C. The PCR product was extracted from 2% agarose gel and purified using the PCR Clean-Up Kit (YuHua, Shanghai, China) according to manufacturer’s instructions and quantified using Qubit 4.0 (Thermo Fisher Scientific, USA). Purified amplicons were pooled in equimolar amounts and paired-end sequenced on an Illumina Nextseq2000 platform (Illumina, San Diego,USA) according to the standard protocols by Majorbio Bio-Pharm Technology Co. Ltd. (Shanghai, China). The raw sequencing reads were deposited into the NCBI Sequence Read Archive (SRA) database (Accession Number: PRJNA1402311).

Raw FASTQ files were demultiplexed using an in-house Perl script, and then quality-filtered by fastp (https://github.com/OpenGene/fastp, version 0.19.6) and merged by FLASH (http://www.cbcb.umd.edu/software/flash, version 1.2.11) with the following criteria: (i) The reads were truncated at any site receiving an average quality score of <20 over a 50 bp sliding window, and the truncated reads shorter than 50 bp were discarded; reads containing ambiguous characters were also discarded; (ii) only overlapping sequences longer than 10 bp were assembled according to their overlapped sequence. The maximum mismatch ratio of overlap region is 0.2. Reads that could not be assembled were discarded; (iii) samples were distinguished according to the barcode and primers, and the sequence direction was adjusted, exact barcode matching, 2 nucleotide mismatch in primer matching.

The resulting high-quality sequences were then clustered into operational taxonomic units (OTUs) using UPARSE 7.1 with a 97% sequence similarity level. The most abundant sequence for each OTU was selected as a representative sequence. The OTUs assigned to spike-in sequences were filtered out and reads were counted. A Standard curves (based on read counts versus spike-in DNA copy number) for each sample were generated, and the quantitative abundance of each OTU in a sample was determined. The taxonomy of each OTU representative sequence was analyzed by RDP Classifier version 2.2 against the fungal ITS reference database (UNITE version 9.0) using a confidence threshold of 0.7. To account for differences in sequencing depth across samples, the OTU abundance table was normalized using a rarefaction approach prior to downstream statistical analyses.

### 2.4 Data analysis

Sequencing reads were normalized by rarefaction to an equal depth across all samples prior to downstream analysis to account for uneven sequencing effort and to ensure unbiased community comparisons. Following sequencing, Origin 2021, Excel 2021, and SPSS 20.0 were used for data organization and statistical analysis. Soil physicochemical characteristics, fungal community diversity indices, and community composition were analyzed using one-way analysis of variance (ANOVA), and group differences were compared using Tukey’s test. The alpha diversity (Chao index, Shannon index, and Simpson index) of the fungal community was calculated using the aforementioned OTU table were characterized. Beta diversity, calculated based on the Bray-Curtis distance to assess differences in microbial fungal structure among samples, was statistically tested for variability within treatments using PERMANOVA with 999 permutations (Adonis function) and visualized via Principal Coordinates Analysis (PCoA) plots. Redundancy analysis (RDA) was employed to reveal the relationships between the fungal community and key environmental factors. Statistical significance of the overall model and individual environmental factors was validated via 999 permutations (conducted in R software using the vegan package). Using FUNGuild v1.0 software, the potential ecological roles of soil fungi were examined. Spearman correlation analysis was used to examine the relationship between fungal functions and soil physicochemical properties.

Co-occurrence network analysis was constructed by Spearman correlations using the “corr.test” function in the “psych” R package. To enhance the ecological relevance and stability of the network, only OTUs with relative abundances > 0.01% of the total fungal OTUs were retained for construction, thereby excluding rare taxa that may introduce stochastic variations. The correlation matrix was filtered using a threshold of |r| > 0.6, which was determined based on random−matrix−theory methods. This threshold not only retains statistically significant correlations but also focuses on strong ecological associations, reducing the inclusion of weak, potentially spurious links. In the resulting network, each node in the network represents a fungal taxon, and the edges signify correlations among different nodes. Using the igraph package in R-4.3.1, a set of topological coefficients of each network, including network diameter, modularity, graph density, average degree, and average path length, were calculated to assess the complexity of the network. The network was visualized using Gephi 0.9.2.

## 3 Results

### 3.1 Soil physicochemical properties in different vegetation types

**Figure 2:**
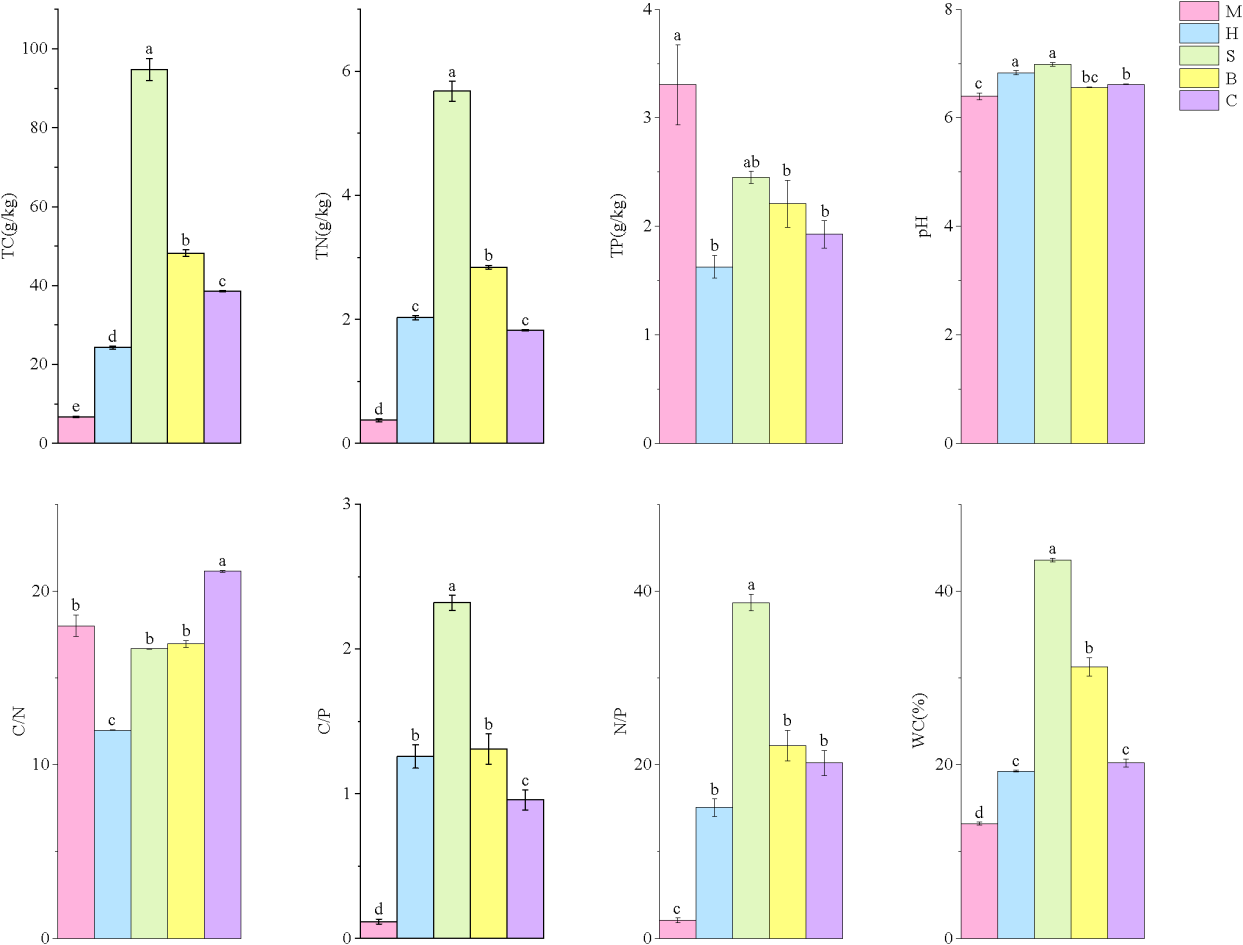
Soil physicochemical properties in different vegetation types. Data are represented as mean ± standard error (n = 3). Different lowercase letters (a, b, c, d) above the bars within the same factor indicate statistically significant differences (*p* < 0.05) based on Tukey’s honestly significant difference *post-hoc* test.

During the transition from the M community to the C community, soil TC, total nitrogen TN, pH, N/P ratio, C/P ratio, and moisture content all exhibited a trend of initially increasing followed by a decrease. Moreover, soil TC (94.75 g/kg), TN (5.68 g/kg), and WC (43.59%) in the S community were significantly higher than in other vegetation types (*p* < 0.05, **Figure 2**). The C/N ratio showed an opposite trend with vegetation type, with the H community exhibiting a significantly lower C/N ratio (11.98) than other vegetation types (*p* < 0.05). While TP exhibited a trend of decreasing, it was highest in the M community (3.30 g/kg) compared to other vegetation types.

### 3.2 Soil fungal community diversity in different vegetation types

**Figure 3:**
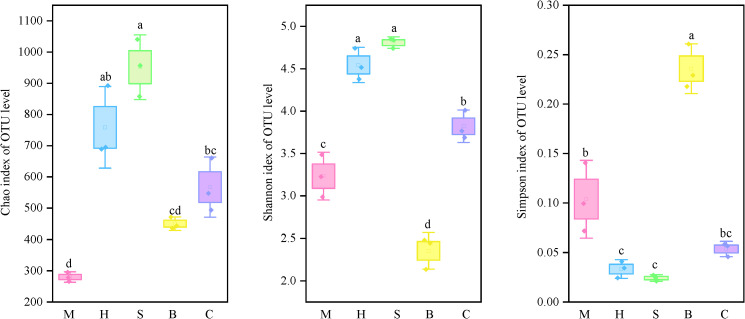
Soil microbial diversity indices in different vegetation types. Data are represented as mean ± standard error (n = 3). Different lowercase letters (a, b, c, d) above the bars within the same factor indicate statistically significant differences (*p* < 0.05) based on Tukey’s honestly significant difference *post-hoc* test.

Across all vegetation types, the Chao and Shannon indices of soil fungal alpha diversity showed an N-shaped trend, peaking in the S community and falling in the B community (*p* < 0.05). The Simpson index, on the other hand, exhibited the reverse pattern, peaking in the B community and falling in the S community (**Figure 3**). The results indicate that there were significant differences in the diversity and richness of soil fungal communities in different vegetation types (*p* < 0.05).

**Figure 4:**
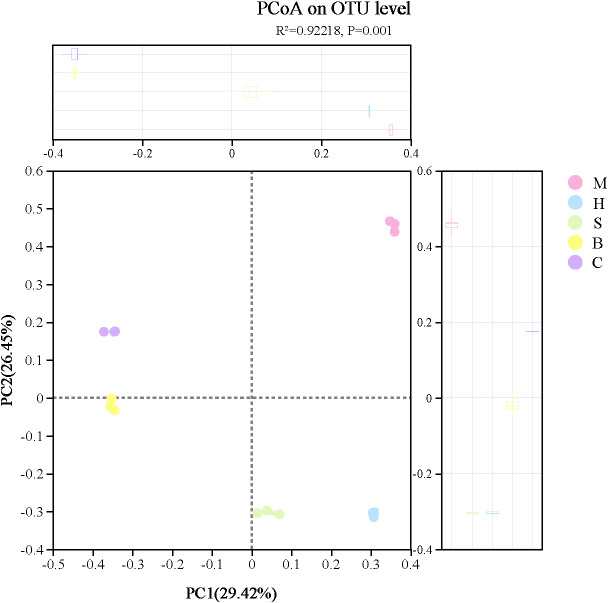
PCoA analyses of soil microbial communities in different vegetation types.

PCoA based on Bray-Curtis dissimilarities revealed a significant separation of fungal community structures among the different vegetation types (PERMANOVA test with 999 permutations: R² = 0.92, *p* = 0.001; **Figure 4**). The first two principal coordinates (PC1 and PC2) explained 29.42% and 26.45% of the total variance, respectively, cumulatively accounting for 55.87%. In the ordination space, samples exhibited clear separation by vegetation type. The M community formed a distinct cluster in the first quadrant, whereas the C community clustered in the second quadrant; both groups were located on the positive side of PC2. Conversely, the B community was situated in the third quadrant, and the S and H communities co-clustered in the fourth quadrant, all on the negative side of PC2. This spatial separation indicates distinct soil fungal community structures across vegetation types. Specifically, the proximity of the S and H communities suggests a higher degree of compositional similarity between them compared to other vegetation types.

### 3.3 Fungal community composition and abundance in different vegetation types

High-throughput sequencing yielded 1413798 valid fungal sequences, which were aggregated into 2821 operational taxonomic units (OTUs).

**Figure 5:**
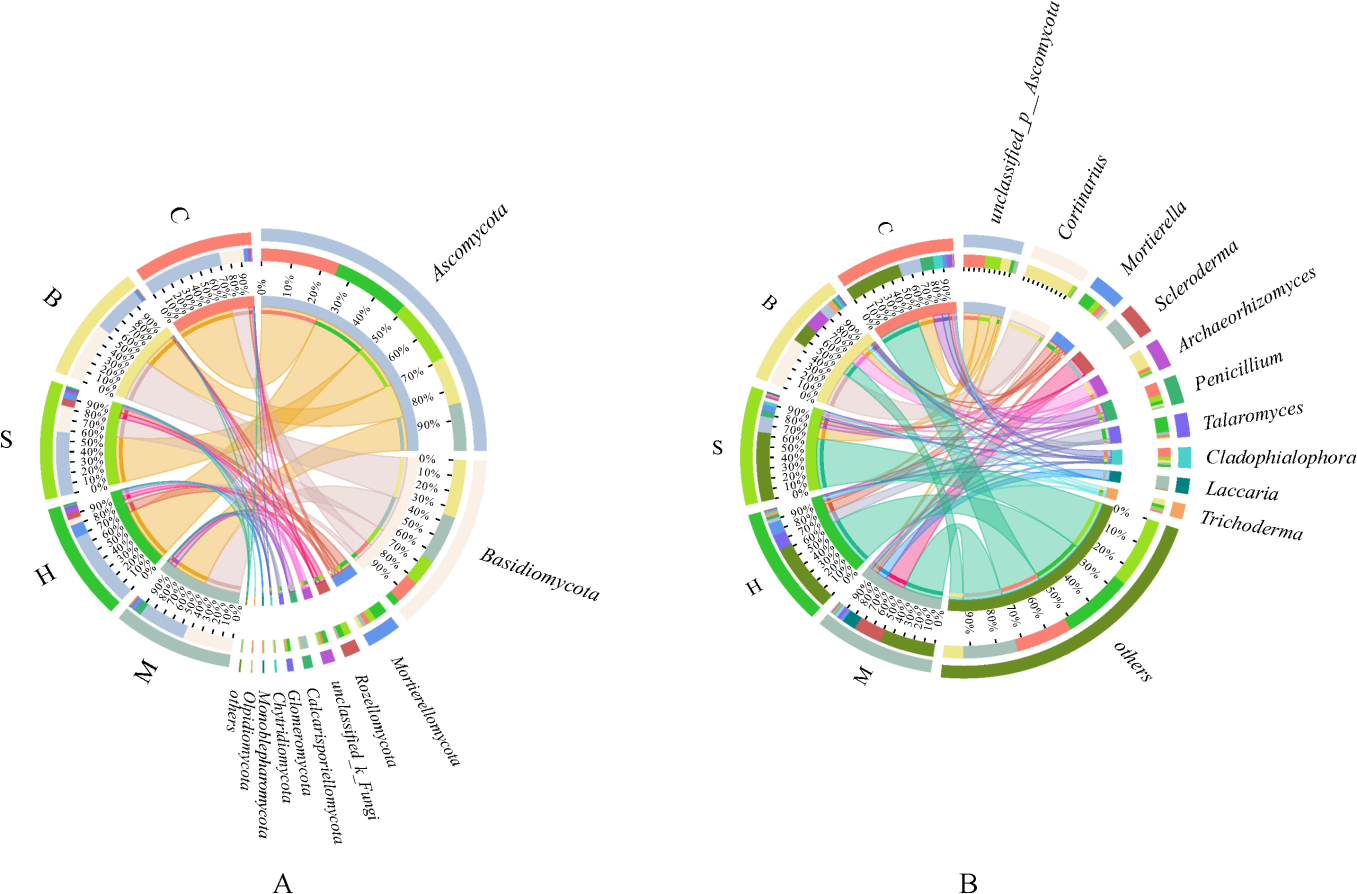
Soil fungal community compositions at the phylum **(A)** and genus **(B)** levels.

At the phylum level (**Figure 5A**), the dominant soil fungal communities were *Ascomycota* (43.39%-71.54%), *Basidiomycota* (5.36%-53.21%), and *Mortierellomycota* (2.38%-11.95%). Ascomycota predominated in both the H (69.25%) and C (71.54%) communities, with the highest abundance observed in C, which was significantly greater than in the S (58.55%), B (43.48%), and M (43.39%). *Basidiomycota* was the most dominant in the B (53.21%), with its abundance following the order: B > M > S > C > H. *Mortierellomycota* was the most dominant in the H community (11.95%), with a relative abundance significantly higher than in other types (*p* < 0.05).

At the genus level (**Figure 5B**), considerable compositional differences were observed across vegetation types. The dominant genera of fungal communities were *unclassified_p_Ascomycota*, *Cortinarius*, *Mortierella*, and *Scleroderma*. *Unclassified_p_Ascomycota* was the most dominant in the C community (19.49%), with its abundance following the order: C > S > B > H > M. *Cortinarius* showed the highest relative abundance in the B community (44.29%), but in M, H, and C, its relative abundance was less than 1%. *Mortierella* exhibited its highest relative abundance in the H community (11.57%), which was significantly greater than that in the other types (*p* < 0.05). In contrast, *Scleroderma* reached its maximum relative abundance in the M community.

### 3.4 Correlation analysis between fungal communities and soil environmental factors

**Figure 6:**
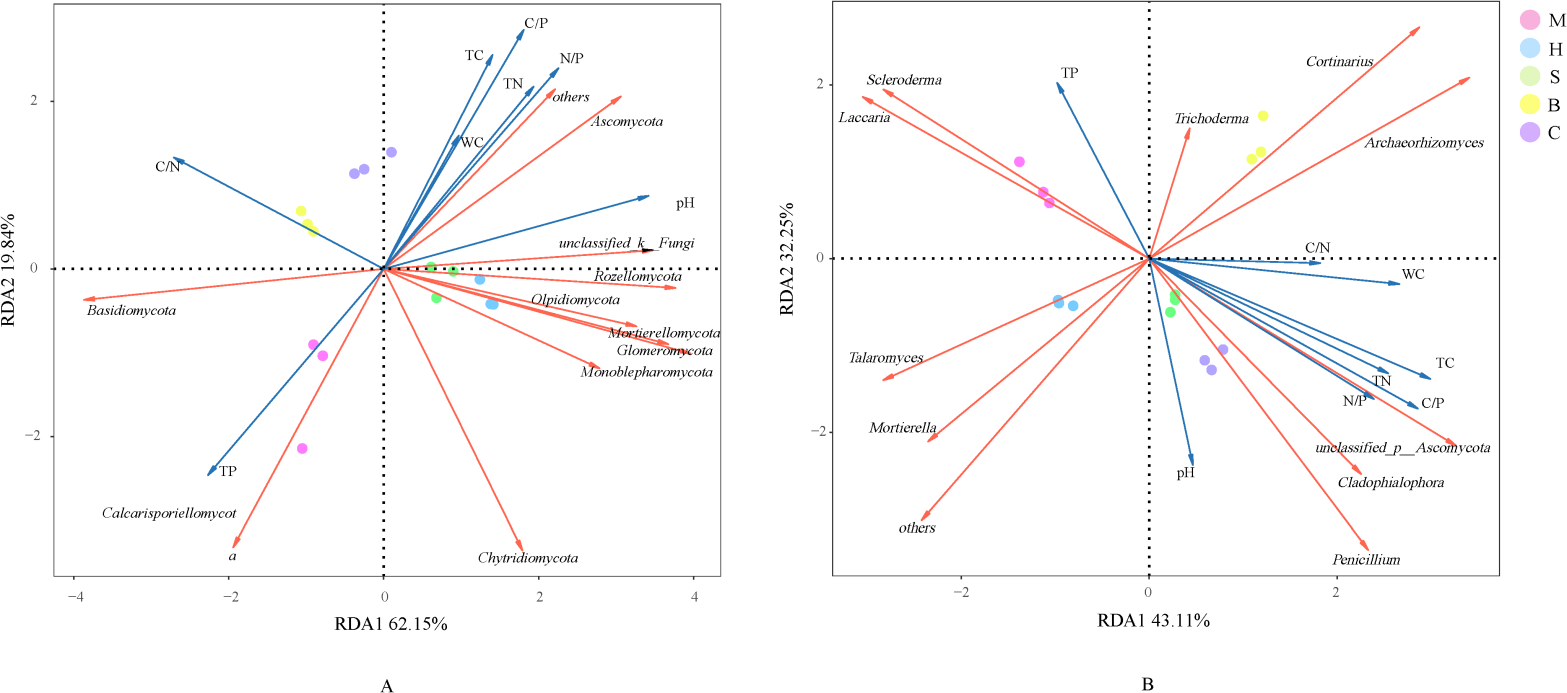
Redundancy analysis (RDA) of soil fungal communities across different vegetation types at the phylum **(A)** and genus **(B)** levels.

Redundancy analysis (RDA) was constructed with log-transformed fungal phylum relative abundance data as the response variable and selected soil physicochemical properties (TP, pH, C/N ratio, N/P ratio, etc.) as explanatory variables. At the phylum level (**Figure 6A**), the first two axes collectively explained 81.99% of the variance in fungal community composition (RDA1: 62.15%; RDA2: 19.84%). TP (R^2^ = 0.749, *p* = 0.001), pH (R^2^ = 0.750, *p* = 0.001), C/N ratio (R^2^ = 0.530, *p* = 0.001), and N/P ratio (R^2^ = 0.415, *p* = 0.043) were identified as significant drivers of community structure (*p* < 0.05). Notably, *Ascomycota* relative abundance correlated positively with N/P ratio and pH, but negatively with TP. Conversely, *Basidiomycota* was positively associated with TP and C/N ratio, and it was significantly enriched in the M community.

At the genus level (**Figure 6B**), the first two RDA axes explained 43.11% and 32.25% of the variance, respectively, yielding a cumulative explanation of 75.36%. The C/P ratio (R² = 0.501, *p* = 0.014) was identified as the most influential driver of fungal community structure among the measured environmental factors. The relative abundances of *Cladophialophora* and *Penicillium* were significantly positively correlated with the C/P ratio, N/P ratio, and pH, and both genera were predominantly enriched in the S and C communities. In contrast, *Mortierella* and *Talaromyces* were primarily associated with the H community. *Scleroderma* and *Laccaria* were mainly found in the M community and showed a significant positive correlation with TP. Finally, *Archaeorhizomyces* and *Cortinarius* were predominantly restricted to the B community.

### 3.5 Co-occurrence network analysis of soil fungal communities in different vegetation types

**Figure 7:**
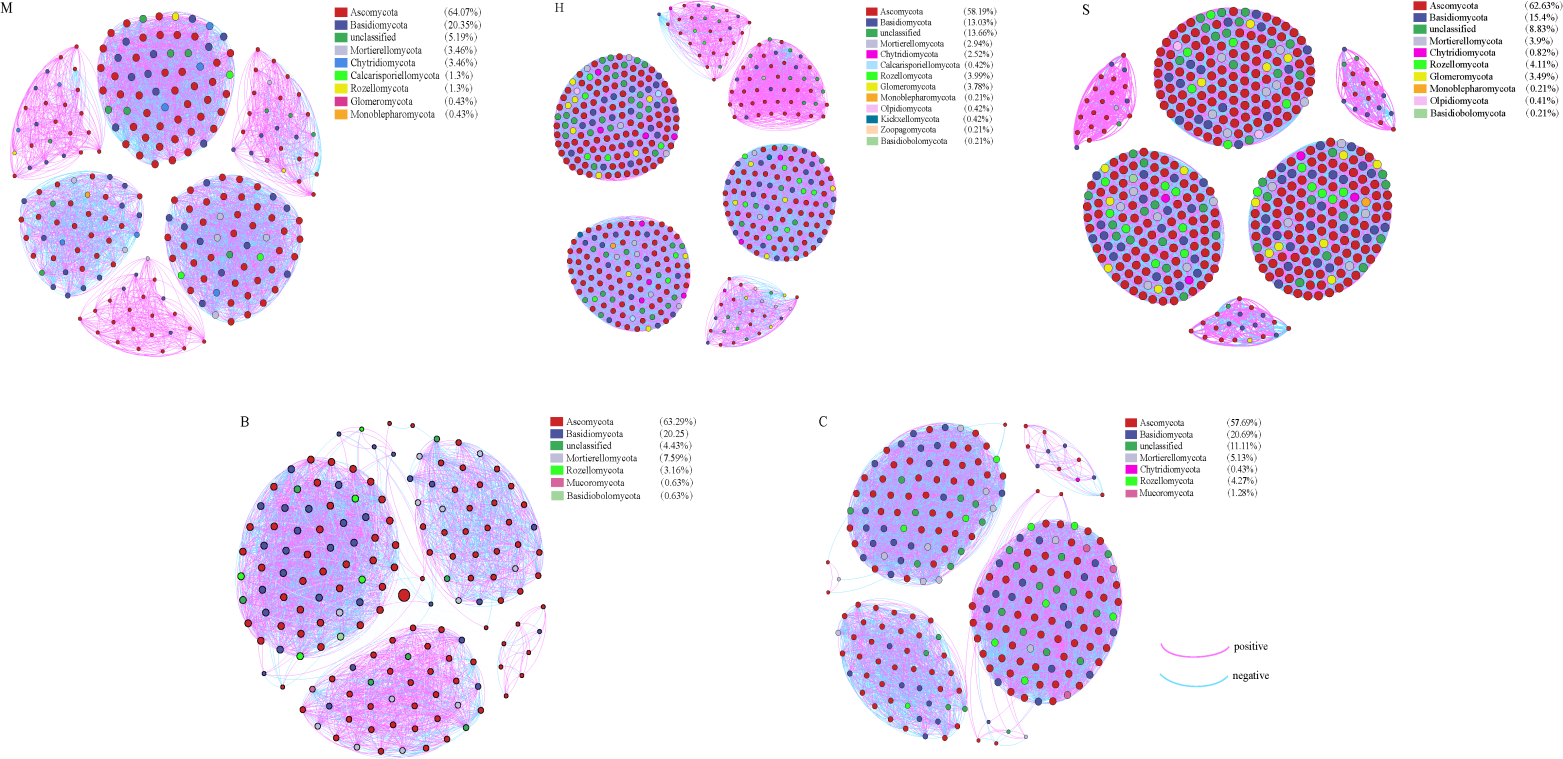
Co-occurrence network analysis and topological characteristics of soil fungal communities. Significant correlations (Spearman’s |r| > 0.6, p < 0.05) exist between taxa. The size of nodes corresponds to the degree of connectivity, and colors distinguish different phyla. Pink edges represent positive correlations, while blue edges represent negative correlations. **(M)** Moss. **(H)** Herb. **(S)** Shrub. **(B)** Broadleaf Forest. **(C)** Coniferous-broadleaf mixed forest.

As shown in **Figure 7**, co-occurrence network analysis at the OTU level revealed distinct topological structures across vegetation types and identified key fungal taxa. Ascomycota and Basidiomycota were identified as the main keystone taxa, despite dynamic fluctuations in their relative abundances across vegetation types. In particular, the C community had the lowest relative abundance of Ascomycota (57.69%), while the M community had the greatest (64.07%). Basidiomycota, on the other hand, was comparatively lower in the H community (13.03%), but it was greater in the M (20.35%) and C (20.69%) communities.

Network topology parameters further uncovered pronounced structural disparities in fungal communities across vegetation types (**Table 2**). Vegetation types S, H, and C formed highly complex, tightly interconnected networks, each characterized by an average path length and network diameter of 1. This topological configuration implies that all nodes were either directly connected or linked via at most one intermediary, suggesting a high potential for efficient propagation of ecological information or resource exchange. Among these, network S exhibited the largest scale (487 nodes, 30,790 edges) and strongest connectivity (average degree: 126.45). Network C displayed the highest density (0.293), and its greater proportion of positive correlations (53.55%) indicated a community structured primarily by synergistic biotic interactions. In contrast, network M featured a distinct “high-modularity, low-connectivity” architecture. It yielded the highest modularity index (0.74) across all vegetation types, accompanied by a notably high proportion of positive correlations (60.63%). This pattern points to a community organized into multiple ecologically discrete modules, predominantly governed by cooperative interactions. Network B, by comparison, exhibited a sparsely connected and structurally simplistic topology. It contained the fewest nodes and edges, alongside the longest average path length (1.72) and largest network diameter (2) across all groups. These metrics align with weak direct interactions among fungal taxa and reflect the lowest overall network complexity relative to other vegetation-associated networks.

**Table 2:** Topological properties of the network of the fungal community in different vegetation types.

Vegetation type	Node	Links	Average degree	Average path length	Network density	Positive edges	Negative edges	Network diameter	Modularity
M	231	4999	43.28	1	0.19	60.63%	39.37%	1	0.74
H	476	25370	106.60	1	0.22	53.22%	46.78%	1	0.68
S	487	30790	126.45	1	0.26	50.44%	49.56%	1	0.68
B	158	3469	43.91	1.72	0.28	58.60%	36.87%	2	0.59
C	234	7987	68.26	1	0.29	53.55%	47.45%	1	0.60

### 3.6 Fungal function prediction in different vegetation types

The ecological functions of fungal communities were predicted using the FUNGuild database. After filtering unassignable fungi, nine trophic modes were identified based on the top 10 relative abundances (**Table 3**). Symbiotroph was the most prevalent trophic mode in the B community (49.46%), showing the highest relative abundance among all vegetation types. Saprotroph was the predominant trophic mode in the H (32.97%), S (29.49%), and C (32.06%), although its relative abundance varied across these vegetation types. Furthermore, the relative abundance of Saprotroph-Symbiotroph was significantly higher in the H (11.72%) community than in other vegetation types, while Pathotroph was significantly less abundant in the B (0.37%) and C (0.99%) communities compared to the rest. In contrast, Pathotroph-Symbiotroph (8.59%) reached its highest relative abundance in the C community.

**Table 3:** Relative abundance of trophic types in soil fungal communities of different vegetation types.

Trophic type	M(%)	H(%)	S(%)	B(%)	C(%)
Symbiotroph	44.88 ± 3.44 ^a^	16.40 ± 1.29 ^b^	18.56 ± 1.11 ^b^	49.56 ± 0.66 ^a^	18.02 ± 1.04 ^b^
Saprotroph	21.50 ± 3.18 ^b^	32.97 ± 1.75 ^a^	29.49 ± 1.68 ^ab^	29.4 ± 0.68 ^ab^	32.06 ± 0.83 ^a^
Saprotroph_Symbiotroph	4.16 ± 0.30 ^bc^	11.72 ± 0.92 ^a^	5.63 ± 1.02 ^b^	2.10 ± 0.62 ^c^	3.95 ± 0.29 ^bc^
Pathotroph_Saprotroph	4.21 ± 1.42 ^a^	5.49 ± 0.72 ^a^	3.28 ± 0.27 ^ab^	1.05 ± 0.10 ^b^	4.12 ± 0.48 ^a^
Pathotroph_Saprotroph_Symbiotroph	9.03 ± 1.99 ^a^	8.37 ± 0.70 ^a^	7.01 ± 0.31 ^ab^	1.11 ± 0.08 ^c^	3.62 ± 0.54 ^bc^
Pathotroph_Symbiotroph	0.62 ± 0.18 ^b^	0.09 ± 0.02 ^b^	0.11 ± 0.01 ^b^	0.09 ± 0.01 ^b^	8.59 ± 0.32 ^a^
Pathotroph	2.28 ± 0.37 ^b^	3.75 ± 0.20 ^a^	3.24 ± 0.41 ^ab^	0.37 ± 0.05 ^c^	0.99 ± 0.12 ^c^
Saprotroph_Pathotroph_Symbiotroph	0 ± 0 ^a^	0 ± 0 ^a^	0 ± 0 ^a^	0 ± 0 ^a^	0.04 ± 0.03 ^a^
Pathogen_Saprotroph_Symbiotroph	0 ± 0 ^a^	0.01 ± 0.01 ^a^	0 ± 0 ^a^	0 ± 0 ^a^	0.02 ± 0.01 ^a^

Different lowercase letters (a, b, c, d) above the bars within the same factor indicate statistically significant differences (*p* < 0.05) based on Tukey’s honestly significant difference *post-hoc* test.

**Figure 8:**
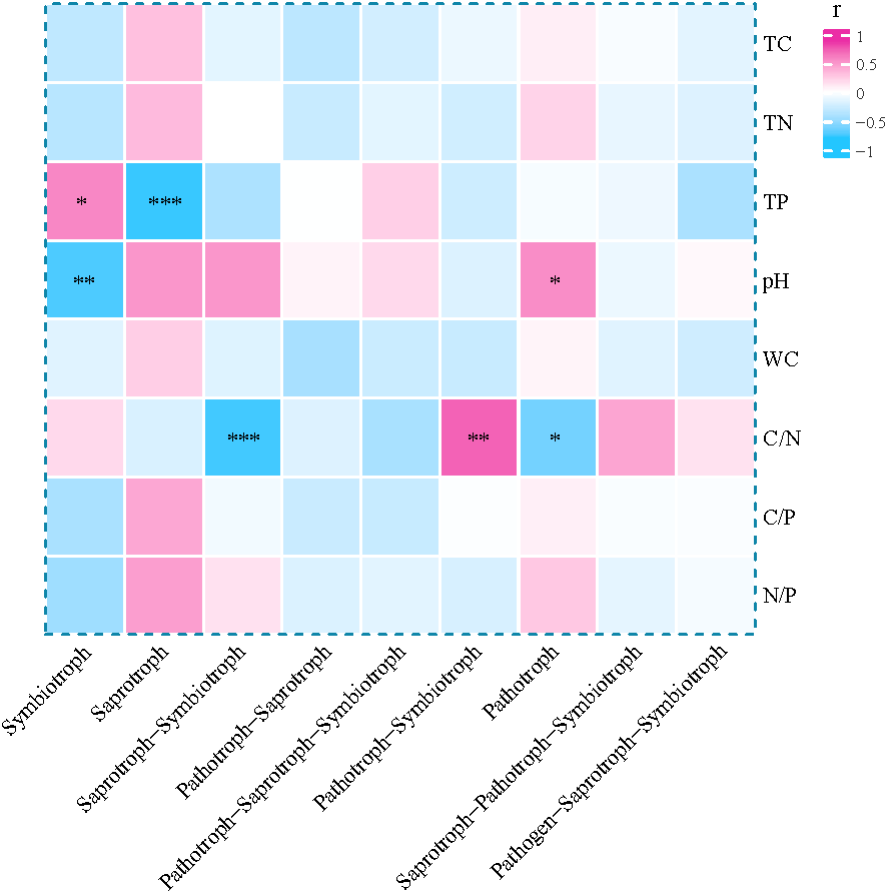
Correlation between environmental factors and fungal functions. *p < 0.05, **p < 0.01, ***p < 0.001.

Correlation analysis (**Figure 8**) between fungal trophic types and soil physicochemical properties revealed that the relative abundance of Symbiotroph was significantly positively correlated with TP (*p* < 0.05) and significantly negatively correlated with pH (*p* < 0.01). The soil C/N ratio showed a strongly significant negative correlation with the Saprotroph-Symbiotroph (*p* < 0.001) and a significant positive correlation with the Pathotroph-Symbiotroph (*p* < 0.01). In contrast, Pathotroph exhibited a significant positive correlation with pH (*p* < 0.05) and a significant negative correlation with the C/N ratio (*p* < 0.05).

## 4 Discussion

### 4.1 Diversity and composition of soil fungal communities

Based on the data obtained in this study, the results suggest that soil fungal alpha diversity (as indicated by the Chao and Shannon indices) is significantly higher in shrub communities (S) than in moss (M) or broadleaf forest (B) communities within this volcanic habitat. These diversity patterns appear to be closely linked to variations in key soil properties, including soil total carbon, and total nitrogen content. In accordance with the general understanding that environmental conditions shape fungal community composition (Shen et al., 2015) and that alpha diversity serves as an indicator of species abundance and richness (Li et al., 2022), our observations in this volcanic ecosystem align with broader ecological principles. Similar patterns have been reported by Miao et al. (2025), who observed that shrub communities promote litter input and root exudation, which in turn are associated with enhanced soil structure and nutrient retention. These improved soil conditions are consistent with a favorable environment for microbial growth and reproduction (Dai et al., 2022) and may contribute to creating diverse niches for fungi, which may reduce interspecific competition and consequently support higher alpha diversity of the fungal community (Yang et al., 2022). Furthermore, such conditions can sustain a greater variety of functional fungi (e.g., decomposers and symbionts), thereby being associated with enhanced overall community complexity (Xu et al., 2021). This is analogous to findings in other volcanic contexts, where ash deposition has been noted to influence fungal diversity through modifications of soil physicochemical properties (Xie et al., 2024). These findings reinforce the ecological importance of soil properties in shaping fungal diversity, and suggest that comparable vegetation–soil–microbe dynamics may also occur in other volcanic systems. Thus, the elevated alpha diversity in shrublands can be interpreted as a direct biological response to the improved soil habitat they engineer.

Vegetation type is a key determinant of soil fungal community characteristics (Cheng et al., 2024). In this study, *Ascomycota* (43.39%-71.54%) and *Basidiomycota* (5.36%-53.21%) are the dominant fungi communities of soil fungi in Wudalianchi. The volcanic lava platform is characterized by less water and good permeability, which are consistent with the characteristics of Basidiomycetes and Ascomycetes that prefer soil permeability (Ni et al., 2021). *Ascomycota*, in particular, are known to exhibit a competitive advantage in nutrient-limited environments (Zou et al., 2024), a pattern further supported by prior research (Siles and Margesin, 2017; Adamczyk et al., 2019). *Cortinarius*, *Mortierella*, and *Scleroderma* emerged as dominant fungal genera, with broad environmental adaptability and the ability to form mutualistic symbioses with plants. These traits are crucial for sustaining soil nutrient cycling and ecosystem stability (Truong et al., 2024). The genus *Mortierella* not only thrives in oligotrophic environments, a trait that facilitates its survival and proliferation (Hansen et al., 2019), but also exhibits notable stress tolerance (Ochoa-Henriquez et al., 2024). In broadleaf forests, the relative abundance of *Cortinarius* was significantly higher than in other vegetation types, a result consistent with the findings reported by Tuo et al. (2022). This distribution aligns with the specific habitat requirements of ectomycorrhizal fungi, as broadleaf forests generally feature deeper root systems, higher soil permeability, and more stable soil structure than moss, herb, or shrub communities, and typically experience less soil disturbance (Lann et al., 2024). These conditions provide suitable habitats and abundant organic substrates for ectomycorrhizal fungi such as *Cortinarius* (Lindahl et al., 2021). The highest relative abundance of *Scleroderma* was observed in the moss (M) community, a pattern consistent with the findings of Chen et al. (2023). This is primarily attributed to its role in enhancing phosphorus availability through the secretion of enzymes and organic acids, which improves the soil microenvironment of the moss community, promotes the accumulation of organic matter and phosphorus, and thereby sustains nutrient cycling within this community (Zhang et al., 2024).

### 4.2 Co-occurrence network analysis of fungal communities

Microbial network analysis serves as an effective tool for investigating interactions within microbial communities under changing environmental conditions (Tao et al., 2018). The complexity and topological characteristics of soil fungal co-occurrence networks reflect the spatial distribution patterns of microorganisms across different vegetation types while elucidating species coexistence relationships (Freitas et al., 2024). Li et al. (2024) discovered that *Ascomycota* and *Basidiomycota* often serve as keystone species in these networks, playing a central role in maintaining network stability and functionality—a finding that aligns with the results of the present study. Their remarkable capacity for organic matter decomposition, broad ecological adaptability, and capacity for symbiosis significantly underpin community stability and ecosystem functioning (Qu et al., 2024).

Abrego et al. (2020) found that in forest ecosystems, microbial communities exhibit an interaction pattern dominated by symbiotic associations, complemented by competitive interactions, which is consistent with the results of this study. Symbiosis reduces direct resource competition and mitigates interspecific exclusion by promoting resource complementarity, functional division of labor, and positive feedback mechanisms (Tedersoo et al., 2020), thereby contributing to ecosystem stability. Furthermore, fungal communities in these systems form complex network architectures (Tian et al., 2025a). Within nutrient-limited volcanic soils, the complexity of fungal co-occurrence networks varies significantly across vegetation types (Yang H.L et al., 2024). Fungal community diversity drives the enhancement of topological structure and the stabilization of key community functions (Tian et al., 2025b), ultimately shaping soil microbial communities with increased resilience to disturbance. This underscores that soil fertility and heterogeneity are primary drivers of microbial interaction complexity. This study found that the fungal co-occurrence network in shrub communities exhibits more nodes, more connections, and a higher average degree compared to other communities, indicating tighter connectivity and more robust interaction patterns. These structural characteristics of the network are closely related to their unique habitat conditions. Shrub communities predominantly occur in complex microtopographic areas such as lava fissures and folds (Liu et al., 2025), where microtopography promotes the accumulation of soil, moisture, and litter—a process that not only results in complex and uncertain distribution patterns of soil properties but is also critical during initial pedogenesis (Bonnet et al., 2023). This mechanism may contribute to the more robust nodes and connectivity pathways observed in fungal−bacterial interaction networks within shrub communities (Mao et al., 2014).

### 4.3 Functional analysis of soil fungal communities

Soil fungal trophic types are closely associated with vegetation types (Hou et al., 2024). While Whitaker et al. (2015) reported that nutrient enrichment generally promotes saprotrophs while inhibiting symbiotrophs, our study revealed a divergent pattern under high TP conditions. Elevated soil P content increases the availability of inorganic nutrients, which reduces plants’ reliance on nutrients derived from organic matter decomposition. Consequently, this diminishes the resource base and competitive advantage of saprotrophs, compressing their ecological niche and leading to reduced diversity–a result consistent with our findings (Yang B. et al., 2024). In the M community, higher TP content slows the decomposition of soil organic matter (Clausing et al., 2021), thereby reducing the substrate availability for saprotrophs. Concurrently, the microbial community structure shifts toward symbiotroph dominance. These combined factors further reduce the competitiveness of saprotrophs. Consequently, the relative abundance of saprotrophs in the M community is significantly lower than in other vegetation types (Ma et al., 2021). This illustrates how specific soil chemical factors (e.g., P availability) can override general patterns to shape functional guild distributions.

## 5 Conclusions

The development of distinct vegetation types on volcanic lava plateaus makes the study of plant-soil-fungal interactions ecologically significant. In nutrient-poor, highly permeable substrates, fungal communities are dominated primarily by *Ascomycota* and *Basidiomycota*. Soil TP, pH, and N/P ratio are key environmental drivers influencing fungal community structure across different vegetation types. During ecosystem restoration, shrubs serve as a critical vegetation type. This microenvironment enhances soil total carbon and nitrogen content, improves soil properties, promotes the formation of highly interconnected fungal interaction networks, and regulates the dynamic balance between symbiotic and saprophytic fungi. Consequently, it effectively boosts the system’s nutrient cycling capacity and ecological stability. This study establishes an integrated analytical framework linking vegetation type, soil properties, and fungal communities, providing insights into the assembly mechanisms of microbial communities in extreme volcanic habitats. Due to sample size limitations, the detection of subtle ecological effects remains constrained. Future research should further validate and refine the proposed “vegetation-soil-microorganism” synergistic restoration mechanism by increasing replicates, expanding spatiotemporal scales, conducting cross-habitat comparisons, and integrating multi-omics approaches.

## Data Availability

The original contributions presented in the study are publicly available. This data can be found here: NCBI Sequence Read Archive, accession PRJNA1402311.
