# Supplementary material for: Analysis of fungal community structure and co-occurrence networks across vegetation types in volcanic lava habitats
Source: Front Fungal Biol. 2026 Feb 24;7:1760883. doi: 10.3389/ffunb.2026.1760883 (PMC12971694; doi:10.3389/ffunb.2026.1760883)
Supplement: Supplementary file 1 [file DataSheet1.pdf]

# *Supplementary Material*

## 1 Supplementary Data

### 1.1 Supplementary Tables

**Supplementary Table 1.** Soil fungal community compositions at the phylum level. Data are represented as mean ± standard error (n = 3). Different lowercase letters in the same index indicate significant differences ($p < 0.05$).

| Fungal Phyla | M(%) | H(%) | S(%) | B(%) | C(%) |
|---|---|---|---|---|---|
| *Ascomycota* | 43.39±4.47 c | 69.25±1.20 a | 58.55±0.88 b | 43.48±0.92 c | 71.54±0.66 a |
| *Basidiomycota* | 44.70±2.79 b | 5.36±0.10 d | 24.34±1.37 c | 53.21±1.09 a | 21.73±1.12 c |
| *Mortierellomycota* | 3.58±0.09 bc | 11.95±0.91 a | 5.99±1.18 b | 2.38±0.71 c | 3.97±0.29 bc |
| *Rozellomycota* | 0.06±0.02 b | 5.20±1.00 a | 6.55±0.28 a | 0.42±0.14 b | 0.40±0.06 b |
| *unclassified_k__Fungi* | 0.97±0.59 bc | 4.17±0.38 a | 1.82±0.14 bc | 0.43±0.08 c | 2.01±0.73 b |
| *Calcarisporiellomycota* | 6.80±1.66 a | 0.05±0.02 b | 0±0 b | 0±0 b | 0±0 b |
| *Glomeromycota* | 0.02±0.02 c | 2.91±0.30 a | 1.54±0.14 b | 0±0 c | 0±0 c |
| *Chytridiomycota* | 0.47±0.21 b | 0.51±0.01 a | 0.20±0.04 ab | 0.02±0 c | 0.06±0.04 c |
| *Monoblepharomycota* | 0.02±0 b | 0.12±0.02 b | 0.67±0.16 a | 0±0 b | 0±0 b |
| *Olpidiomycota* | 0±0 a | 0.30±0.21 a | 0.14±0.05 a | 0±0 a | 0±0 a |
| *others* | 0.01±0 a | 0.19±0.07 a | 0.19±0.09 a | 0.06±0.03 a | 0.28±0.18 a |

**Supplementary Table 2.** Soil fungal community compositions at the genus level. Data are represented as mean ± standard error (n = 3). Different lowercase letters in the same index indicate significant differences ($p < 0.05$).

| Fungal Genera | M(%) | H(%) | S(%) | B(%) | C(%) |
|---|---|---|---|---|---|
| *unclassified_p__Ascomycota* | 2.60±1.53 e | 3.26±0.40 d | 14.60±1.70 b | 8.85±1.18 c | 19.49±0.58 a |
| *Cortinarius* | 0.11±0.03 c | 0.32±0.06 c | 3.02±0.16 b | 44.29±1.62 a | 0.21±0.14 c |
| *Mortierella* | 3.55±0.11 bc | 11.57±0.91 a | 5.33±0.98 b | 2.05±0.62 c | 3.72±0.25 bc |
| *Scleroderma* | 25.70±4.50 a | 0.02±0.01 b | 0.31±0.14 b | 0±0 b | 0±0 b |
| *Archaeorhizomyces* | 0.55±0.15 b | 0.60±0.15 b | 2.19±0.34 b | 17.29±3.20 a | 2.89±0.99 b |
| *Penicillium* | 0.65±0.15 d | 2.91±0.11 c | 5.44±0.33 b | 2.31±0.15 c | 11.76±0.60 a |
| *Talaromyces* | 2.61±0.29 b | 13.98±1.43 a | 0.51±0.04 bc | 0.38±0.04 c | 1.99±0.12 bc |
| *Cladophialophora* | 1.90±0.81 bc | 1.43±0.18 c | 3.20±0.23 b | 2.14±0.36 bc | 8.27±0.64 a |
| *Laccaria* | 12.11±1.56 a | 0.21±0.05 b | 0.01±0 b | 0.01±0.01 b | 0.01±0 b |
| *Trichoderma* | 1.52±0.40 a | 2.82±0.24 a | 1.77±0.30 a | 5.24±3.32 a | 0.77±0.09 a |
| *others* | 48.70±1.98 b | 62.86±0.79 a | 63.63±2.35 a | 17.42±1.99 c | 50.88±1.25 b |
